# Professionals' views on providing personalized recurrence risks for de novo mutations: Implications for genetic counseling

**DOI:** 10.1002/jgc4.1910

**Published:** 2024-06-25

**Authors:** Alison C. Kay, Jonathan Wells, Anne Goriely, Nina Hallowell

**Affiliations:** ^1^ MRC Weatherall Institute of Molecular Medicine, Radcliffe Department of Medicine University of Oxford Oxford UK; ^2^ NIHR Biomedical Research Centre Oxford UK; ^3^ The Centre for Personalised Medicine University of Oxford Oxford UK; ^4^ Clinical Genetics St. Michael's Hospital Bristol UK; ^5^ Ethox Centre and Wellcome Centre for Ethics and Humanities University of Oxford Oxford UK

**Keywords:** mosaicism, precision medicine, prenatal testing, recurrence risk

## Abstract

When an apparent de novo (new) genetic change has been identified as the cause of a serious genetic condition in a child, many couples would like to know the risk of this happening again in a future pregnancy. Current practice provides families with a population average risk of 1%–2%. However, this figure is not accurate for any specific couple, and yet, they are asked to make decisions about having another child and/or whether to have prenatal testing. The PREcision Genetic Counseling And REproduction (PREGCARE) study is a new personalized assessment strategy that refines a couple's recurrence risk *prior* to a new pregnancy, by analyzing several samples from the parent–child trio (blood, saliva, swabs, and father's sperm) using deep sequencing and haplotyping. Overall, this approach can reassure ~2/3 of couples who have a negligible (<0.1%) recurrence risk and focus support on those at higher risk (i.e. when mosaicism is identified in one of the parents). Here we present a qualitative interview study with UK clinical genetics professionals (*n* = 20), which investigate the potential implications of introducing such a strategy in genetics clinics. While thematic analysis of the interviews indicated perceived clinical utility, it also indicates a need to prepare couples for the psychosocial implications of parent‐of‐origin information and to support their understanding of the assessment being offered. When dealing with personalized reproductive risk, a traditional non‐directive approach may not meet the needs of practitioner and client(s) and shared decision‐making provides an additional framework that may relieve some patient burden. Further qualitative investigation with couples is planned.

**What is known about this topic**

De novo mutations (DNMs) cause developmental disorders in ~1 in 295 live births. In ~10% of families, one of the parents is mosaic for the DNM which is associated with a recurrence risk of up to 50%. Until recently, it was not possible to identify these high‐risk couples and all families receive the same population average recurrence risk of ~1%–2%. This uncertainty can lead to anxiety and/or unnecessary invasive prenatal testing for reassurance in future pregnancies, when in fact, for the majority the recurrence risk is negligible.

**What this paper adds to the topic**

The personalized risk assessment offered by PREGCARE, a novel genomic test, adds clarity to DNM recurrence risk management and supports reproductive decision‐making *prior* to a new pregnancy. Implementation of the strategy in a clinical setting will require additional training for practitioners about communication of the testing approach and the interpretation of personalized recurrence risks for couples considering further pregnancies.

## 1 INTRODUCTION

Integration of new genomic technologies in clinical practice requires a multifaceted approach focusing on responsibilities around the sharing of genetic information and practitioner–patient relationships (Juengst et al., 2016; van den Boer‐van & Maat‐Kievit, 2001), including effective communication of risk (Biesecker, 2018; Clarke & Thirlaway, 2011; Dragojlovic et al., 2020; Horne, 2017; Stoll et al., 2018). This aspect is particularly relevant in implementation of so‐called personalized strategies which necessitates parallel advances in how practitioners relay the information, support understanding, and deliver counseling to enable informed choice for patients (Feiler et al., 2017; Gaitskell, 2017; Hollands et al., 2016; Horne, 2017; Marteau et al., 2010). There are also many current drivers of new genomic strategies (commerce, government targets, and patient groups), and clear evidence of patients' benefit is also needed (Turnbull et al., 2024).

The birth of a child with a serious clinical disorder, often involving complex learning disabilities and/or severe physical impairment and/or shortened lifespan, is a life‐changing event for all family members (Boardman & Clark, 2022). When a causative gene alteration is identified in their child, but is not found in either parent, they are told it is most likely to have been caused by a de novo mutation (DNM)—that is one‐off mutational event—occurring within a single gamete from one of the parents (egg or sperm) or early in the child's own embryonic life. However, a DNM can sometimes arise at an earlier timepoint in the embryonic development of one of the parents and be present in multiple gonadal cells (“gonadal mosaicism”), leading to a risk of recurrence in future pregnancies. In this case, the recurrence risk could be as high as 50%.

Due to this uncertainty, practitioners provide a population‐average recurrence risk estimate of ~1%–2%, even though for the vast majority of couples, the risk is negligible because the mutational event occurred in a single gamete. Some couples may not feel reassured by the population average risk. An unexpected event has already happened to them once and they may decide to forego further pregnancies or seek reassurance in the form of an invasive intervention such as prenatal testing in a future pregnancy.

This study explores UK practitioner perspectives on the wider implications of introducing a new personalized strategy which aims to provide personalized recurrence risk assessment to parents of children affected by genetic conditions caused by a “de novo mutation” (DNM).

### 1.1 Personalizing recurrence risk for de novo mutations

**FIGURE 1:**
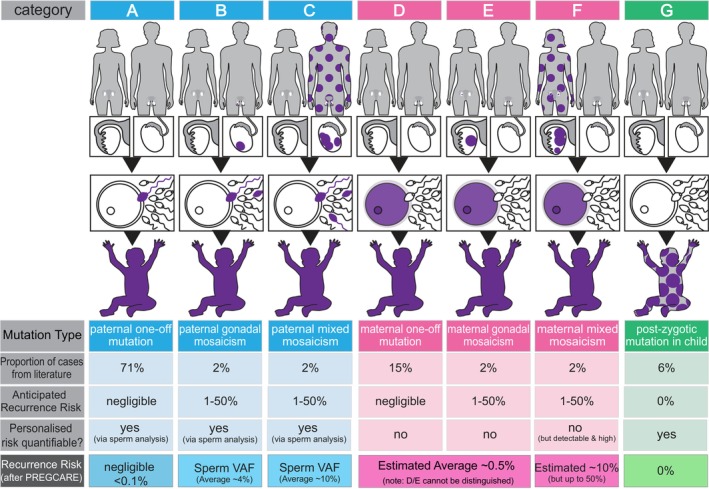
PREGCARE outcomes. Overview of the PREGCARE strategy for DNM recurrence risk assessment and summary of key results. By establishing the origin (paternal (blue), maternal (pink) or post‐zygotic (proband, green)), and the timing of the mutational event (purple color indicates mutant cells), the PREGCARE research study was able to stratify the majority of families into one of seven different categories that are associated with widely different recurrence risks (see “anticipated recurrence risk” on the figure). The proportion of cases in each category can be estimated using data from the literature (Rahbari et al., 2016). Four of the seven categories (i.e. categories B, C, F, and G) involve mosaic presentations and can be identified using deep sequencing of the tissues collected from the mother–father–child family trio. Furthermore, analysis of a sperm sample for paternal cases allows personalized quantification of the risk to another pregnancy (i.e. via direct measurement of the variant allele frequency (VAF) of the DNM in the paternal semen sample). By singling out these mosaic families, the remaining (mosaic‐negative by deep sequencing) categories (A/D/E) have a reduced risk of recurrence estimated to be ~0.1% (>10‐fold reduction over the generic 1%–2% risk) (see Bernkopf (2023) for details). Analysis of the DNM parent‐of‐origin via long‐read haplotyping provides further refinement of the risk for the mosaic‐negative categories. This offers reassurance to the majority of families because Category A (one‐off paternal, 71% of cases) is associated with a negligible recurrence risk—estimated to be <0.1% depending on the limit of detection (sensitivity) of the specific DNM custom assay. Note that for a proportion of families enrolled in the PREGCARE study (~15%), the parent‐of‐origin of the DNM could not be resolved (not represented here). The last row of the figure describes an overview of the refinement of personalized risk afforded by the PREGCARE strategy; note that categories D/E cannot be distinguished from one another because it is not possible to access maternal oocytes; and therefore, the risk for a mosaic‐negative DNM of proven maternal origin is estimated to be reduced modestly (~2–8×) compared to the population generic risk. Moreover, the risk associated with Category F (maternal mixed mosaicism) cannot be quantified but should be considered to be “high.” Modeling indicates the risk for Category F to be on average ~ 10% (considering VAF in maternal blood <15%). For further details on the PREGCARE strategy, results, risk estimates, and references, see Bernkopf (2023). Figure adapted from Bernkopf (2023).

To improve the information available to couples and practitioners, the PREGCARE study (PREcision Genetic Counseling And Reproduction) used ultra‐deep sequencing of several tissues (blood, saliva, buccal swabs, urine, and sperm) from 60 parent–child trios to stratify families into one of seven possible categories associated with widely different recurrence risks (summarized in Figure 1). Importantly, to provide individualized reassurance to families in whom mosaicism was not detected by deep sequencing, this strategy requires determination of the parent‐of‐origin of the DNM (Bernkopf, 2023). This is because the vast majority (~80%) of DNMs originate during adult spermatogenesis, and in these cases, analysis of a sperm sample provides a direct quantification of the risk in future pregnancies. For cases of proven maternal origin, the risk can be estimated but not quantified as oocytes cannot be accessed directly. The risk for a DNM of proven maternal origin is estimated to be reduced modestly (~2–8×) compared to the population generic risk (Bernkopf, 2023).

Although PREGCARE offers potential clinical utility and reassurance to most families regarding recurrence risks in a future pregnancy (Bernkopf, 2023; Kay et al., 2023), the personalized nature of the information obtained presents new challenges for genetic counseling. In particular, the determination of parent‐of‐origin which is not usually discussed in counseling for DNMs may be problematic—even if it is an information routinely disclosed to couples for inherited conditions.

### 1.2 Genetic counseling and consent

Currently, routine genetic counseling supports the consent process using a non‐directive approach in the provision of information, avoiding guiding clients with their practitioner opinion or by suggesting a particular action (Arribas‐Ayllon et al., 2009; Bredenoord et al., 2010; Elwyn et al., 2000; Hens et al., 2013; Hertig et al., 2014; Hines et al., 2010; Leefmann et al., 2017; Townsend et al., 2012). However it has been questioned whether non‐directiveness in practice is achievable, for example because of the unequal nature of the encounter between client and practitioner (Arribas‐Ayllon et al., 2011; Clarke, 1991; Hallowell, 1999a; Kessler et al., 1984; Kirklin, 2007; O'Doherty, 2009; van den Boer‐van & Maat‐Kievit, 2001; Weil, 2003). Similarly, framing effects can unintentionally steer the decision‐making process (Davey et al., 2005; Kahneman & Tversky, 2000; Kirklin, 2007; van der Steen et al., 2019).

Additionally, in the UK, genetic counseling is increasingly provided outside of genetics clinics (Brittain et al., 2017). In other specialities, a shared decision‐making approach is more familiar to practitioners, in which they educate, support, and guide their patients to informed decisions considered value‐congruent (Birch et al., 2019; Kalsi et al., 2019). In the context of reproductive health, midwives and obstetricians have reported experiencing slippage in non‐directiveness, at least partly due to patient requests for more direction (Brittain et al., 2017; White et al., 2020; Williams et al., 2002).

The present qualitative study considered the wider implications of PREGCARE, a novel personalized reproductive risk information for genetic counseling provision. It investigated the experiences and perspectives of medical geneticists and genetic counselors experienced in providing recurrence risk counseling following a DNM‐related diagnosis.

## 2 METHODS

Ethics approval was obtained from the Medical Sciences Interdivisional Research Ethics Committee (MS IDREC) and the University of Oxford Central University Research Ethics Committee (R77884/RE001), in accordance with the University's procedures for obtaining ethical approval of all research involving human participants.

The research team comprised a practicing genetic counselor, a genetic counseling researcher, a medical sociologist, and molecular geneticist. Additionally, most authors have previous experience using qualitative research approaches and all possess genetic health expertise.

Participants were recruited via the membership of the British Society of Genetic Medicine (BSGM) and the Association of Genetic Nurses and Counselors (AGNC), inviting participation of Medical Geneticists and Genetic Counselors with experience in providing reproductive genetic counseling for DNMs. Participants were recruited until no new issues or themes were mentioned during interviews. In total, medical geneticists (MG) and registered genetic counselors (GCr) from 15 National Health Service Trusts across the UK were recruited. Of these, 12 had direct experience of referring couples into the original PREGCARE study and all had experience of counseling couples about DNM recurrence risk and prenatal testing options. Among the medical geneticists (*n* = 14), there were nine women and five men. Of the genetic counselors interviewed (*n* = 6), there was one man, reflecting the gender composition of the profession (Kopesky et al., 2011).

Semi‐structured interviews (duration ~60 min) were conducted by AK, a postdoctoral researcher with training in genetic counseling. Recorded interviews took place remotely via MS Teams between February and June 2022, approximately 10 months after the last family was enrolled into the PREGCARE research study (March 2021). The semi‐structured interview guide was used to gather information on each participant's current approach to DNM counseling, including their views on the current practice of generic recurrence risk provision, how they explain DNM recurrence risk, and any challenges encountered in their counseling practice. They were also asked about their views on the range of PREGCARE outcomes (see Figure 1) and, if they had direct experience with PREGCARE, how the couple responded during counseling. Finally, they were invited to reflect on the practicalities and implications of introducing PREGCARE into clinical practice.

The data generated were managed in NVivo, a qualitative data analysis software package, and all transcribed data were initially read several times by AK. Reflexive thematic analysis was used to analyze the data (Braun & Clarke, 2006, 2019). This “Big Q” qualitative approach is grounded within the qualitative paradigm and involves using inductive coding to identify consistent thematic elements in the data and possible relationships between them (Braun & Clarke, 2023). Data and themes were discussed by AK and NH at regular intervals to ensure analytic rigor and agreement on the final elements, their interrelationships, and mapping. Field notes were a useful means or reflective practice throughout. Standards for Reporting Qualitative Research (SRQR) guidelines were observed (O'Brien et al., 2014).

## 3 RESULTS

**FIGURE 2:**
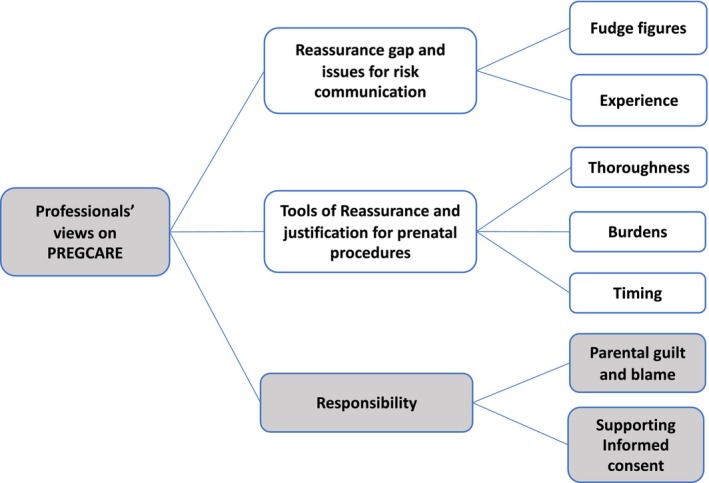
Coding for thematic analysis. The focus of this study relates to the theme of responsibility, while the two other themes were reported in a previous study by Kay et al. (2023).

A number of themes reflecting practitioners' experiences and views were identified. These included the *reassurance gap and communication challenge for mosaicism* and also *tools of reassurance*, which were reported in a separate paper (Kay et al., 2023). The theme of *responsibilities* for practitioners when counseling about recurrence risk was also identified and is the focus of this paper (see Figure 2).

Firstly, practitioners acknowledged the potential for psychosocial impact on couples in receiving a personalized recurrence risk assessment, in particular feelings of guilt and blame. In relation to this, they reflected on their own responsibility as practitioners to deliver information in a way that would not fuel these reactions, while acknowledging the concept of mosaicism and the PREGCARE testing procedure are complex and would be challenging to explain to most couples. On the other hand, they emphasized the challenge to remain non‐directive and provide full information for consent.

### 3.1 Implications for parental guilt and/or blame

The practitioners interviewed in this study told us that when PREGCARE results confirmed the DNM had originated during the child's embryonic development rather than in the parents (post‐zygotic DNM) (see Figure 1, category G), this would be helpful to couples because “it's something that they didn't have anything to do with” [MG13]. This scenario, for which there is no risk of DNM recurrence, nor parental blame, was seen as relieving uncertainty and providing reassurance. However, the six alternative PREGCARE categories (Figure 1, categories A–F) were noted as more challenging—with the potential to escalate feelings of blame and guilt for some couples—because they required determination of the parent‐of‐origin of the DNM in the affected child and the recognition that “…‘it came from me’, rather than ‘We don't know where it came from’, it's quite a different thing for people to get their heads around” [MG08]. Currently, before undergoing genetic testing for their child, couples are told various potential inheritance scenarios could be identified. When the results do not indicate the presence of the child's genetic variant in either parent, they are told the condition has been caused by a de novo change that was likely a “one‐off” event and only in rare cases could it be due to parental mosaicism. The couple may feel some relief and would be in: “…blissful ignorance about not attributing the source of the pathogenic variant to either parent” [MG15]. But the PREGCARE strategy requires knowledge of the origin of the DNM in the affected child to establish the risk for future pregnancies and this new information could be upsetting: “I think that was quite a shock for her to know that it had come from her when previously we said, ‘It's de novo’. So, I think changing that information was upsetting for her.” [MG08] (see Table 1 MG10).

**TABLE 1:** Additional quotations.

MG10	But I can definitely see harm in just being more definitive about which germ cell had the pathogenic variants. Yeah, I think it will be very difficult to know how different couples would react to that, but I can definitely see real harm and feelings of guilt and blame
MG11	I think that there will be some ethnic groups where discussing this will be very difficult because of their cultural and religious backgrounds. And certainly, if you look at some of those groups, where this information might be stigmatising for them as a couple and definitely stigmatising if it's the man or the woman, and they will be very private about that information
MG03	…at least it gives them information that they can then, you know, think about their range of options etc. You know they might want to visit sperm donation…
MG15	I think it's paternalistic and inappropriate to not pursue the pathway because you're worried that there may be a result that…that increases their anxiety
MG02	…it needs careful handling and an explanation before you have it and an explanation after you've had it, what it actually means for you and what difference it will make when you're if you choose another pregnancy
GCr07	I think it's really important, you want to be non‐directive…It's really important that they know all of the options available to them
MG04	I think and hope that the skill you use in counseling can help make people understand that it isn't a blame or a fault thing
GCr20	…as someone who advocates for the couple, I would want as much information as they want to receive

The risk of blame and/or guilt was partly seen as stemming from a lack of current preparedness of couples regarding the complex biology of mosaicism, but also for fathers regarding their increased paternal age‐related risks and the greater potential for them to be the parent‐of‐origin of the DNM:

I think the downside is, what we've never done is discuss parent‐of‐origin and that whole guilt thing that …even though we know that the risk is…it's more likely that it's paternal, particularly if it's an older father, we've generally tended to say, ‘It could be in the ovaries or the testes’ and we don't attribute blame, whereas of course there is that possibility through PREGCARE that needs to be explored. [MG12]

Some interviewees reflected further that discussions about maternal age and risks to the fetus are common in clinic and public discourse (e.g. the increased risk of Down's Syndrome in older mothers), “…whereas men, I think, don't realise that the risk of problems goes up as they get older. It's a bit of a shock to them.” [MG12].

Given this lack of preparation, some practitioners deliberated as to whether fathers would be disproportionately impacted by receiving new parent‐of‐origin information, although generally there was some ambivalence on this. For example, a practitioner who participated in PREGCARE noted: “I don't recall either of the two mothers where it was of maternal origin were upset by that but the dad, when there was the high‐risk case, was very upset.” [MG04]. Some mentioned that fathers would carry greater self‐blame and were concerned that personalizing recurrence risk could bring the “…risk of Dad throwing himself off a bridge…I'm being dramatic but you know.” [MG16], whereas others said that in other types of inheritance, with a few exceptions, generally men exhibit less guilt than mothers at being identified as parent‐of‐origin:

I don't feel that they would stew on it like a mother would, I think. I don't know that I would necessarily warn that there is a high chance it is going to be them. [MG18]

Parent‐of‐origin information was thought to require particular consideration when offering PREGCARE testing to couples from communities practicing consanguineous marriage. Independently of the mode of inheritance, interviewees noted that “… in some cultures there's an element of ‘other blame’ You know, there is an assumption that it is Mum's fault” [MG16], something they did not want to exacerbate. Also, if the onward risk management options after providing PREGCARE results would be unacceptable to couples on religious or cultural grounds, then the clinical utility would be lower and “… these families probably wouldn't go forward with this type of testing…” [MG18] (see Table 1 MG11).

For any couple, regardless of cultural or religious background, obtaining personalized reproductive information after a DNM‐related diagnosis of a child might have a disruptive effect on their relationship with each other. As GCr07 said, pregnancy and difficult pregnancies, in particular, are often seen as a “shared journey.” They went on to say:

This happened to both of you and you're both in it together and actually, when you split it out and say, ‘Well, but one person is the cause’, well, that's quite painful. I think that would be really on my mind. [GCr07]

Defining one person in the couple as being the originator of their child's serious condition, no matter how unknowingly, has the potential to disrupt that support mechanism and negatively impact their relationship:

It may change the dynamics between the parents…now they know it's from one of them. [MG15]

However, practitioners did not regard concerns around parent‐of‐origin information and their potential to fueling guilt or blame to be a reason to forego providing information to couples and support evidence‐based personalized decision (see Table 1 MG03; MG15; GCr07):

I can see, you know, it can be challenging obviously dealing with feelings of guilt with those parents, but I don't feel like that would be an overriding reason not to do it. I don't feel like that would cause more harm than actually the benefits of knowing and being able to kind of change management accordingly. [GCr19]

I don't know what parents will do with that information, but I do think it might be useful for them to make decisions, even if it's one that is unusual, or even if we need to make adjustments in the way we counsel… [MG13]

The potential to provide personalized reproductive recurrence risk following the birth of a child with a DNM using PREGCARE was seen as offering “an answer finally…closure in a way” [MG05]. They said knowing the parent‐of‐origin, although not reassuring in itself, would lead to new risk management options and refined counseling.

### 3.2 Implications for supporting decision‐making and consent

Practitioners, both those who took part in PREGCARE and those who did not, considered a “*careful*” [GCR07] consent process as a good means of identifying and filtering out those couples who would find PREGCARE helpful and those for whom it would raise anxiety or cause other issues (see Table 1 MG02; MG04):

I think if you've counselled that the risk can go down as well as up, I think that's fair and I think that if they understand that and consented to that, then you're not ambushing them with something they didn't want. [MG15]

We offered them the option of PREGCARE …and actually the couple chose not to find out. They'd rather have not known where it might have come from to sort of keep the…to take the blame out of it, I guess, and the guilt out of it, as they might see it… [MG03]

However, despite their desire to obtain fully informed consent ahead of a personalized recurrence risk assessment, both medical geneticists and genetic counselors expressed doubts as to whether practitioners would be able to secure couples' full understanding. This is because mosaicism is a complex process and is hard to understand—even for health professionals—and the details of the genetic testing are often unfamiliar to couples.

Interviewees told us that in practice mosaicism, especially somatic mosaicism, is given a brief and simplified explanation during consultations because it involves “incredibly complex pieces of information” [MG10] that can be “really difficult to explain” [MG15]. They thought the “level of genetic literacy and the complexity” [GCr07] would make germline mosaicism and recurrence risk even harder to explain, thereby potentially undermining the informed consent process. Additionally, the range of possible PREGCARE scenarios was likely to add to the complexity of pre‐test counseling. They said all possible scenarios would require an explanation because there is uncertainty as to which would be relevant for a specific couple (Figure 1 summarizes the different outcomes). Practitioners noted that an added complication arose for maternally originating DNMs, as PREGCARE would only provide a new “refined” risk estimate rather than a risk quantification—because the oocytes are inaccessible to direct analysis. Overall, because of this remaining uncertainty, they were less sure how to counsel couples for DNMs of maternal origin (Categories D–F on Figure 1).

Interestingly, the practitioners also saw a challenge in being non‐directive or in being perceived as non‐directive in the face of complex and psychosocially demanding information. They said patients may perceive authority in information provided by a healthcare professional, even if not their intention, and this may influence their decision‐making:

I think the other thing to say is that if you offer somebody further investigation, they might feel like they have to take it up. So even if you offer it really non‐directively and say, ‘If you're interested, there is this service. We're happy to recruit you or give you more information’, they may feel that if they don't take that up that they're not doing everything they can to prevent a recurrence. [GCr20]

Alternatively, some practitioners said they were wrestling with an element of tension in their responsibility to be considerate of psychosocial consequences of the information they deliver and considered the possibility of withholding potentially worrying information from very anxious couples:

I just think when you isolate it to one parent or the other, I think there's careful conversations to be had there, and whether or not that actually makes it back to the clinic is a question that I've not considered, you know, whether I would accidentally withhold that information but just give the result in a very neutral way that tells us that the recurrence risk is low but, you know, that's difficult. [MG16]

…part of me did think ‘Ooh do we want people to know that’ but of course I think where it is relevant – which clearly is in terms of defining risk and giving people information for their future pregnancies. [GCr17]

## 4 DISCUSSION

Participants in this study expressed a responsibility to consider clinical utility against the psychosocial impact of receiving personalized information, consistent with the widely held goal of the relief of guilt and shame in genetic counseling (Kessler et al., 1984). Of particular concern was the identification of parent‐of‐origin information and its potentially negative implications for both mother and father.

In terms of preconception health, most interventions and messages are primarily focused on women (Mello et al., 2020). Mothers are also more prominent in parental accounts of responsibility (Dimond, 2014; Grant et al., 2020; Jackson & Mannix, 2004), with particular concerns and “moral gaze” directed toward smoking and drinking during pregnancy, for which risks have been established (Grant et al., 2020; Ujhelyi Gomez et al., 2022), but also toward a myriad of other potential transgressions for the “shield of safety” around the fetus, often with conflicting or little evidence (Lupton, 2012; McNaughton, 2011).

Due to this gendering of responsibility in the reproductive realm, provision of personalized DNM risk information indicating the mother is mosaic for the DNM variant identified in her child would require skillful genetic counseling to navigate feelings of responsibility or mother blame. However, as was found in a study of families with a child with de novo 22q11 deletion syndrome (*n* = 23), even when couples do not have personalized information, they may draw on gendered responsibility in trying to make sense of the diagnosis (Dimond, 2014).

In this study, some participants thought that the mother blame they perceived to exist in some ethnic cultural groups could potentially make PREGCARE less appealing. Also, if a couple did go ahead and maternal origin was revealed, they wondered if it could be negatively impactful. Firstly, although couples practicing consanguineous marriage were mentioned, it seems likely there was some conflation of marriage practice and ethnic culture in their responses. Additionally, while DNMs occur in all couples equally, the incidence of congenital anomalies in consanguineous families is greater due to recessive inheritance (McRae et al., 2017). A consanguineous couple may experience some relief from a de novo diagnosis that rules out recessive inheritance and the associated high recurrence risk (25%). Whether they would want to refine the recurrence risk by establishing the origin of the DNM was less clear.

Discussion and delivery of personalized information in reproductive healthcare ought to draw on a nuanced understanding of the religious and cultural beliefs of couples (Bentley et al., 2008; Betancourt et al., 2003; Hassan et al., 2020; McGinniss et al., 2018; Warren, 2011). Limited research in this area suggests risk and reproductive decisions in minority groups in the UK and Europe may be more varied and dynamic than first thought (Becker et al., 2015; Shaw, 2011). A study of the British Pakistani community (*n* = 51 couples) found substantial variation in prenatal testing decisions, rather than a blanket rejection, with reproductive choices moving toward risk management over time and with experience, such as having an affected child (Shaw, 2011). Similarly, a 20‐year German study (1993–2012) (*n* = 675 consanguineous pregnancies), which analyzed the risk of major anomalies in the offspring of consanguineous couples living in Berlin, noted a high rate of medical terminations (MTOP) of affected fetuses conceived by couples of Turkish or Eastern Mediterranean origin, in comparison with couples from other Islamic communities, which appears to be indicative of more permissive attitudes toward assessment and MTOP within some Islamic communities (Becker et al., 2015). Whilst not focused on de novo risk, the findings of these studies suggest caution in assuming how couples might react to personalized risk information based on socio‐cultural or ethnic background.

In addition to concerns around exacerbating mother blame, some practitioners thought identifying the father as the parent‐of‐origin of the DNM could also be problematic. They said men may be particularly unprepared to receive this information. This was partly due to the well‐known gendering of responsibility in pregnancy but also to the lack of awareness of the predominance of paternal origin of DNMs or the impact of advanced paternal age on spontaneous genetic disease (Jónsson et al., 2017; Kong et al., 2012). However, despite potentially providing unexpected information for fathers, most interviewees thought a pre‐ and post‐PREGCARE counseling session could help couples understand and adapt to this new information. Of note, explanatory support is already provided for dominant and recessive inheritance patterns.

Reproduction, including infertility, is known to present specific challenges for men and their conception of masculinity and also for health services in terms of how well men are prepared for their reproductive journey (Dolan & Coe, 2011; Hinton & Miller, 2013). Further research is needed to document men's perception and acceptance of recognized paternal reproductive risks relating to DNMs. However, we can draw on existing studies from other potentially psychosocially difficult health circumstances for men, such as male breast cancer risk and prostate cancer treatment side‐effects—both of which bring uncertainty and can disrupt life stories and perceptions of self. Previous evidence has shown that, in these circumstances, men borrow culturally shared storylines around positive masculine identity (Pietilä et al., 2018) to re‐interpret their experience. This “narrative reconstruction” helps them to make sense of their risk or health issue and the ways in which it affects different aspects of their life (Hallowell et al., 2006; Pietilä et al., 2018; Schultze et al., 2020; van der Kamp et al., 2022). In the context of genetic risk information, Hallowell et al. (2006) found that men adapted to information on cancer‐related BRCA1/2 risks in different ways depending on their status as carrier/non‐carrier, in order to present themselves as correspondingly blameless or morally responsible. Men adapted to the status of carrier by using a fatalistic narrative, removing their sense of personal culpability. This enabled them to reconcile their sense of genetic responsibility, self and family (Hallowell et al., 2006).

### 4.1 Issues for supporting patient autonomy in practice

The notion of “genetic responsibility”—decisions about how and whether to act on genetic information (Hallowell, 1999b; Leefmann et al., 2017; Weiner, 2011)—is not only a consideration for individual patients and their families but also important when considering the interactions of the individual with their health practitioner (Koch & Nordahl Svendsen, 2005; Leefmann et al., 2017). However, it is not always easy to define where the boundaries of practitioner responsibility ought to be, as highlighted for example by the debate around the “duty to recontact” when interpretations of genetic risk are updated (Doheny et al., 2018).

The majority of participants in this study talked about the importance of providing the personalized information fully and letting the couple decide what the information would mean for them. Some did consider whether practitioner selectivity—withholding parent‐of‐origin information—might be psychosocially more beneficial for some couples. They were then quick to stress they recognized this as overprotective, and interfering with the principles of autonomy and informed consent. However, reports of selectivity by healthcare professionals when counseling on complex prenatal risks and options have been described before (Hertig et al., 2014), often using a “minimalist” approach to information provision to avoid producing anxiety (Burton‐Jeangros et al., 2013; Hertig et al., 2014).

Alongside concerns about inducing anxiety, practitioners in this study also described the challenge of securing couples' understanding of the testing process, its purpose, benefits and importantly, its limitations, in order to obtain consent. All commented that the phenomenon of mosaicism was generally too complicated and timely to explain in any detail during routine clinical appointments, making it difficult to prepare couples for the PREGCARE testing. This is consistent with other studies relating to the provision of genetic information, with practitioners reporting that gaining understanding for consent cannot always be achieved when the information is complex (Bredenoord et al., 2010; Hens et al., 2013; Schicktanz, 2018).

For example, a range of professionals discussing Preimplantation Genetic Testing for Monogenic Disorders (PGT‐M) and Preimplantation Genetic Screening for aneuploidy (PGT‐A) (*n* = 12) as part of expert panels regarded the complexity of the possible test outcomes beyond the knowledge and intellectual capacity of most couples, creating a stumbling block to autonomous decision‐making (Hens et al., 2013). Similarly, in semi‐structured interviews with professionals working in mitochondrial DNA disease (*n* = 20), participants agreed on the core value of reproductive autonomy but were ambivalent as to whether the nuances of mitochondrial genetics (for which there is also uncertainty in predicting recurrence risk) could be adequately explained to patients which could provide grounds not to offer testing altogether (Bredenoord et al., 2010).

Hence, although understanding is crucial for informed decision‐making (Henneman et al., 2008; van den Boer‐van & Maat‐Kievit, 2001), when introducing personalized reproductive information, such as PREGCARE, the threshold for what needs to be understood may need to be lower than desired. For example, Bernhardt et al. (2015), who explored the process and content of informed consent for genomic sequencing in interviews with US genetic counselors (*n* = 29), found that practitioners tended to focus on common misconceptions, rather than trying to convey scientific principles, technological aspects, or the prospective results and potential for uncertain results (Bernhardt et al., 2015).

Full autonomy when faced with complex concepts and/or information with psychosocial implications may be experienced by some individuals or couples as a moral or psychological burden (Arribas‐Ayllon et al., 2009; Hallowell, 1999a). Moreover, freedom to choose does not necessarily mean knowing what the right choice is (van den Boer‐van & Maat‐Kievit, 2001) and clients may ask for more guidance. Current adherence to non‐directiveness in genetic counseling could mean requests for direct guidance could be met inconsistently by practitioners, especially with mainstreaming, as described in an interview study of Swiss gynecologist–obstetricians and midwives (*n* = 41) (Hertig et al., 2014).

Certainly, clients' hopes or expectations regarding practitioner genetic responsibility can differ to those of the practitioner (Kirklin, 2007; Leefmann et al., 2017). For example, a focus group and interview study (*n* = 15) of Canadian couples consenting to whole‐genome sequencing for their undiagnosed and often acutely ill child concluded that they had unmet decisional needs and providing them with too much information led to overload (Li et al., 2016). Similarly, in an investigation of lay attitudes to predictive testing (*n* = 43), Wohlke and Perry (2021) found that when the participant perceived risk information as complex and/or uncertain, they preferred a more directive physician–patient relationship, recognizing they could not independently understand the genetic information.

Our findings support other studies suggesting that a more overt use of the framework of shared decision‐making (Birch et al., 2019; Elwyn et al., 2000; Hunt et al., 2005) may be beneficial when counseling involves complex information or difficulty with understanding, and implementation or non‐implementation would have significant consequences on the couple's risk management. This would still rely on respect for autonomy of the couple being upheld (Elwyn et al., 2000)—a foundational professional value in genetic counseling (Elwyn et al., 2000; Stoll et al., 2018).

### 4.2 Study limitations

Self‐selecting participation of interviewees was unavoidable, but every effort was made to raise awareness of the study and to conduct the interviews at a suitable time for the participants. Participants were recruited across 15 NHS Foundation Trusts (UK), and they had no prior relationship with the researcher. Consistencies across the participants' responses in this study, and with the existing literature, offer some support for the reliability of our findings. Our results are not intended to be generalizable but rather represent a close examination of the experiences of this opportunity sample. As a group of practitioners with considerable experience in supporting couples, our findings have important implications for genetic counseling associated with personalized reproductive risk assessment for DNMs. Follow‐up research directly involving interviews of couples who have had a child with a DNM is planned because what matters to clients/patients needs to be factored into the development of personalized and targeted interventions (Kent, 2017). It is not yet known how personalized reproductive risk information is perceived by individual couples or if it is beneficial to their decision‐making process in future pregnancies.

## 5 CONCLUSION

There are new responsibilities not only for the receiver but also for the giver when offering more refined, but complex, genetic information (Doheny et al., 2018; Horne, 2017; Moffatt et al., 2014). Practitioners interviewed in this study saw clinical utility in offering PREGCARE—a strategy for personalizing DNM recurrence risk. However, they thought it should be offered by medical genetics specialists in the NHS [Clinical Genetics], rather than being mainstreamed (Kay et al., 2023). Analysis of their responses suggests consideration of the psychosocial implications for each couple will require additional clinic time and practitioner training. Development of new resources to support clinic consultations will also be needed, such as a DNM risk information leaflet and audio‐visual alternatives.

Practitioners were concerned it may be challenging for some couples to achieve understanding of the unfamiliar and complex information associated with personalizing DNM recurrence risk, thereby creating a burden on patients. Incorporation of shared decision‐making could relieve this burden. This would align with a more relational approach to autonomy and allow for the social reality of complex information provision and reproductive decision‐making (Dove et al., 2017; Gómez‐Vírseda et al., 2019; Salema et al., 2019). However, the specifics of this approach in this context require further investigation with couples, particularly regarding the role of health literacy (Elwyn et al., 2000).

## AUTHOR CONTRIBUTIONS

Conceptualization: AG and NH. Funding acquisition: AG. Ethics: JW, NH, and AG. Recruitment: AK, JW, and AG. Data collection: AK. Qualitative analysis: AK and NH. Project administration: AK and JW. Writing—original draft: AK and NH. Writing—review and editing: NH, AG, and AK. Approval: AK, JW, and AG. Author AK confirms full access to all the data in the study and takes responsibility for the integrity of the data and the accuracy of the data analysis. Authors AK and AG gave final approval of this version to be published and agree to be accountable for all aspects of the work in ensuring that questions related to the accuracy or integrity of any part of the work are appropriately investigated and resolved.

## CONFLICT OF INTEREST STATEMENT

Authors AK, NH, AG, and JW declare no conflict of interest.

## ETHICS STATEMENT

Human Studies and Informed Consent: Ethics approval was obtained from the Central University Research Ethics Committee (R77884/RE001) at the University of Oxford and the Medical Sciences Interdivisional Research Ethics Committee (MS IDREC) in accordance with the University's procedures for ethical approval of all research involving human participants. Informed consent was obtained from all participants.

Animal Studies: No non‐human animal studies were carried out by the authors for this article.

## Supporting information


Data S1.

## Data Availability

The data that support the findings of this study are available from the corresponding author upon reasonable request.
